# Cocoa, Hazelnuts, Sterols and Soluble Fiber Cream Reduces Lipids and Inflammation Biomarkers in Hypertensive Patients: A Randomized Controlled Trial

**DOI:** 10.1371/journal.pone.0031103

**Published:** 2012-02-27

**Authors:** Rosa Solà, Rosa M. Valls, Gemma Godàs, Gloria Perez-Busquets, Josep Ribalta, Josefa Girona, Mercedes Heras, Anna Cabré, Antoni Castro, Gema Domenech, Ferran Torres, Lluís Masana, Neus Anglés, Jordi Reguant, Bartolomé Ramírez, Joaquim M. Barriach

**Affiliations:** 1 Unitat de Recerca en Lípids i Arteriosclerosi (URLA), CIBERDEM, Hospital Universitari Sant Joan de Reus, IISPV, Universitat Rovira i Virgili, Reus, Spain; 2 Biostatistics Unit, School of Medicine, Universitat Autonoma de Barcelona, Barcelona, Spain; 3 Statistics and Methodology Support Unit (USEM), IDIBAPS (Hospital Clinic), Biostatistics Unit, School of Medicine, Universitat Autonoma de Barcelona, Barcelona, Spain; 4 La Morella Nuts, Castellvell del Camp, Spain; University of Modena and Reggio Emilia, Italy

## Abstract

**Background:**

Cocoa, mixed with other food ingredients, intake can have beneficial effects on cardiovascular disease (CVD) biomarkers. We compared the effects of 4 cocoa cream products on some of these biomarkers.

**Methods and Findings:**

In this multi-centered, randomized, controlled, double-blind, parallel trial, volunteers (n = 113; age range: 43–65 years) who were pre-hypertensive, stage-1 hypertensive and hypercholesterolemic received one of 4 cocoa cream products (13 g/unit; 1 g cocoa/unit, 6 units/d; 465 Kcal/d) added to a low saturated fat diet for 4 weeks. The groups were: A) (n = 28), cocoa cream considered as control; B) (n = 28), cocoa+hazelnut cream (30 g/d hazelnuts); C) (n = 30), cocoa+hazelnuts+phytosterols (2 g/d); and D) (n = 27), cocoa+hazelnuts+phytosterols+soluble fiber (20 g/d) the patented “LMN product”. Primary outcome measures were BP, LDL-c, apolipoprotein B-100 (Apo B), ApoB/ApoA ratio, oxidized LDL (oxLDL) and high-sensitive C-reactive protein (hsCRP) determined at baseline and post-cocoa cream product intake. Statistical analysis used was ANCOVA or mixed models (in case of repeated measurements), with baseline observation included as a covariate. After 4 weeks, compared to product A, product C reduced LDL-c by 11.2%, Apo B by 8.1% and ApoB/ApoA ratio by 7.8% (P = 0.01). LMN decreased LDL-c by 9.2%, Apo B-100 by 8.5%, ApoB/ApoA ratio by 10.5%, hsCRP by 33.4% and oxLDL by 5.9% (P = 0.01). Surprisingly, even “control” product A reduced systolic BP (−7.89 mmHg; 95%CI: −11.45 to −4.3) and diastolic BP (−5.54 mmHg; 95%CI: −7.79 to −3.29). The BP reductions were similar with the other 3 products. Limitations of the study are that the trial period was relatively short and that a better “BP control” product would have been preferable.

**Conclusion:**

The creams (particularly the LMN) have anti-inflammatory and antioxidant effects in addition to lowering LDL-c, Apo B and ApoB/ApoA ratio. Thus, the soluble fiber effects amplified with sterols (as contained in the cocoa creams) provide new dietary therapeutic perspectives.

**Trial Registration:**

Clinicaltrials.gov NCT00511420

## Introduction

Dietary factors influence plasma lipid levels (such as low-density lipoprotein cholesterol; LDL-c), blood pressure (BP), or other cardiovascular disease (CVD) biomarkers [1]–[4]. Modifications of nutritional components, consumption of specific foods, food additives and supplements are the major dietary approaches to reducing the risk-factors. The most beneficial CVD changes result from: reducing intake of saturated (SFA) and trans fats; an adequate intake of polyunsaturated (PUFA) and increasing the amount of monounsaturated fats (MUFA); fortifying foods with plant stanols or sterols; adding nuts to the diet; increasing the intake of soluble fiber and soy protein; increasing the consumption of oily or fish-derived omega-3 fatty acid or cocoa products and flavonols [5], [6]. However, the effects of incorporating some of these ingredients into a commercial product for consumption as a dietary component are unknown.

In the present study, the hypothesis is that new cocoa product formulations with low saturated fatty acids, moderate sugar content, plus hazelnuts, or phytosterols or soluble fiber in an appetizing cream mixture can induce a reduction of CVD biomarkers when consumed as a dietary supplement.

The aim of our study was to assess the effects of cocoa or cocoa+hazelnut, or cocoa+hazelnuts+phytosterols, or cocoa+hazelnuts+phytosterols+soluble fiber on intermediate metabolic markers of CVD risk. The formulation was tested as part of a calorie-balanced weight-maintaining diet in prehypertensive and stage-1 hypertensive and hypercholesterolemic volunteers.

## Methods

The protocol for this trial and supporting CONSORT checklist are available as supporting information; see Checklist S1 and Protocol S1.

### Participants

The participants were community-dwelling men and women >20 years of age, with prehypertension (systolic BP: 120–139 mm Hg or diastolic BP: 80–89 mm Hg) and stage 1 hypertension (systolic BP: 140–159 mm Hg or diastolic BP: 90–99 mm Hg), LDL-c between 3.35 mmol/L (130 mg/dL) and 4.88 mmol/L (189 mg/dL), and at least one CVD risk factor such as age (men ≥45 years; women ≥55 years), smoking habit, low high density lipoprotein cholesterol (HDL-c) concentration of <1.0 mmol/L (40 mg/dL) and <1.18 mmol/L (46 mg/dL) in men and women, respectively; family history of premature CVD (in male first-degree relative <55 years of age, in female first-degree relative <65 years of age. Exclusion criteria included diabetes mellitus, any chronic disease, current hypolipemic treatment, triglycerides (TG) >3.97 mmol/L (350 mg/dL) in fasting state, body mass index (BMI) >35 kg/m^2^.

### Ethics

Participants provided written informed consent prior to enrolment into the trial and eligibility or exclusion was assessed by the attending physician based on a review of the clinical records, followed by a screening visit. The study was approved by the Clinical Research Ethical Committee (also known as an Institutional Review Board) of the *Hospital Universitari Sant Joan de Reus* and at 3 Primary-Care Centers (Alcover, Vic, Centelles) where participants were recruited and the human experimentation was conducted.The study protocol was in accordance with the Declaration of Helsinki and good clinical practice guidelines.

### Interventions

#### Diet design

The study was a controlled, double-blind, parallel, multi-centered study in which the 4 different cocoa cream products were introduced into a calorie-balanced diet for 4 weeks. There was a prior stabilization period of 2 weeks in which all participants received the cocoa cream product (designated control product A) in order to assess the subject's tolerance to the product. In the stabilization period of 2 weeks duration, the isocaloric intake was the Spanish composition diet [7]; 39% of total energy as fat and, of which, 13% as SFA. In the intervention period of 4 weeks duration, the isocaloric diet prescribed was 35% of total energy as fat of which <7% was SFA, 50% of total energy as carbohydrate, 15% of total energy as protein and <200 mg/d of cholesterol [8], [9].

The nutritional composition of the cocoa cream was taken into account as a basic food component of the diet. The inclusion of cocoa products resulted in a reduction of olive oil as the predominant fat. Also, to reduce SFA intake, items such as sausages, cured or mature cheese, whole fat milk and whole fat yogurt were avoided. However, dietary energy recompense was by increasing intake of carbohydrates provided by bread, pasta and potatoes. Further, we recommended the avoidance of chocolate, products containing stanol esters or sterols, food items or products rich in fiber or omega-3, nuts and soya or soya products. Consumption of legumes was limited to a maximum of 3 times per week.Three-day food records at the beginning and end of the intervention period and a 24 h dietary recall at 2 weeks within the intervention period were used to assess adherence to the recommended diet. The nutritionists underwent training to ensure standardization of procedures and dietary evaluations. The nutrient composition of the diet was calculated using the *Répertoire Géneral Des Aliments* [10].

The trial was conducted in one clinical centre (*Hospital Universitari Sant Joan de Reus*) and 3 Primary-Care Centers (Alcover, Vic, Centelles).

### Cocoa cream products

A low saturated fat, calorie-balanced diet included 6 units (13 g/unit, 465 Kcal/d) of product A: cocoa; or product B: cocoa+hazelnut (30 g/d hazelnuts; 5 g hazelnut in each unit); or product C: cocoa+hazelnuts+phytosterols (2 g/d); or product D: cocoa+hazelnuts+phytosterols+soluble fiber (20 g/d). This last product was designated LMN (patent WO2007063158A2). The quantities of nuts (30 g/d) and soluble fiber (20 g/d) were chosen since these are amounts that have shown anti-CVD effects [5], [6]–[8], [9].

All the products contained approximately 1 g of cocoa solids per unit. All the different cocoa cream products were formulated, manufactured and nutrient evaluated using USDA food composition tables. Individual doses were consumed as snacks or as additions at mealtimes, but without milk or other dairy products.

Compliance was monitored by empty wrapper counting and any non-consumed doses were collected at follow-up clinical visits. We defined non-compliance as a cream consumption of <80%.

Adverse effects were coded according to the MedDra dictionary (version 8.0) and categorized by body system and preferred term.

### Outcomes

Primary outcome measures were BP, LDL-c, apolipoprotein B-100 (Apo B), ApoB/ApoA ratio, oxidized LDL (oxLDL) and high-sensitive C-reactive protein (hsCRP). The variables were determined at baseline (after 2 weeks of stabilization period) and following ingestion of one or other of the 4 different cocoa cream products introduced into a calorie-balanced diet for 4 weeks.

BP was measured by trained personnel. With the subject seated, BP was measured 3 times at 1-minute intervals using an automatic sphygmomanometer (OMRON HEM-907; Peroxfarma, Barcelona, Spain) and the mean value recorded. Weight and height were measured with the participants in indoor clothing without shoes, using calibrated scales and well-mounted stadiometer (Añó Sayol S.A., Huelva, Spain). Bodyweight was measured every 2 weeks and, if the weight varied by >1 Kg, the caloric intake was modified to maintain basal bodyweight for the rest of the study. Waist circumference (WC) was measured midway between the lowest rib and the iliac crest using an anthropometric tape.

In a subgroup of participants, endothelial function was measured using fingertip Peripheral Arterial Tonometry (Endo-PAT) equipment (Hamar Medical, Caesarea, Israel). The Endo-PAT index was calculated as the ratio of the digital pulse volume during reactive hyperemia÷the value at baseline. A value of <1.6 Arbitrary Units (AU) was indicative of endothelial dysfunction.

### Biomarkers

Blood was drawn from each patient following an overnight fast. To reduce intra-individual day-to-day variability, the blood sampling was performed on two separate days at the end of the stabilization period (recorded as “baseline”) and at the end of the intervention period. The blood samples were stored at −80°C in the central laboratory's Biobanc (bancmb@grupsagessa.com) until required for batched analyses.

Lipids and other biomarkers were measured centrally at *Hospital Universitari Sant Joan de Reus* (Catalonia, Spain) or in the URLA laboratories of the *Facultat de Medicina de Reus*.

Total cholesterol (TC), TG, HDL-c, apolipoprotein (Apo) A-1 and Apo B-100, high-sensitive C-reactive protein (hsCRP), interleukin-6 (IL-6) in serum were performed using standard methods in an autoanalyzer (Beckman Coulter-Synchron, Galway, Ireland). LDL-c was calculated by means of the Friedewald formula [11].

Oxidized LDL (OxLDL) by immunoassay (Mercodia AB, Uppsala, Sweden) was measured in EDTA plasma. Vascular cell adhesion molecule type 1 (VCAM-1), intercellular adhesion molecule type 1 (ICAM-1) (R&D Systems, Minneapolis, USA) were measured in citrate plasma using ELISA kits.

### Sample size calculation

This trial was an exploratory study designed to assess the beneficial effects on several CVD biomarkers of cocoa cream additions to the diet. With 25 valid subjects per group, our study was sufficiently powered (i.e. 80%) to detect relevant magnitude of differences such as those observed in a recent meta-analysis [12] for systolic BP of 4.5 mmHg (SD: 10–13 mm Hg) and for diastolic BP of 2.5 mm Hg (SD: 7–9 mm Hg), and a two-sided alpha of 5%. However, we planned to include two additional patients per arm to guarantee an 80% power to detect, as well, a difference of at least 13 mg/dL (0.34 mmol/L) in LDL with a SD of 17 mg/dL (0.44 mmol/L) [13] and a two-sided alpha of 5%. Thus, at least 27 subjects in each arm were recruited.

Further, with 27 valid subjects per group, the study has 80% power to detect a difference of at least 0.34 mmol/L (13 mg/dL) in LDL-c with a SD of 0.44 mmol/L (17 mg/dL), with a two-sided alpha of 5%.

### Randomization sequence generation and implementation

The randomization code was computer-generated random number sequence in gender-stratified blocks of 4 persons each. Center and treatment assignment codes were allocated via an interactive electronic response system administered by the Barcelona Randomization Unit, which took no further part in the study.

### Blinding

The cocoa cream products were manufactured by La Morella Nuts S.A. (Castellvell, Spain) specifically for the trial (Table 1) and all the products were designed to have the same texture and visual characteristics.

**Table 1:** Nutritional composition of each cream (13 g dose).^a^

Content per dose	Product A	Product B	Product C	Product LMN
Energy, Kcal	77	77	73	73
Carbohydrate, g	6.6	5.6	5.4	4.5
Protein, g	0.2	0.9	0.8	0.9
Total fat, g	6.0	6.1	5.8	6.1
Saturated fat, g	1.2	1.3	1.2	1.4
Stearic fatty acid, g	0.3	0.6	0.6	0.5
Monounsaturated, g	3.0	3.6	3.0	3.4
Polyunsaturated, g	1.4	0.9	1.3	1.0
n-6, g	3.0	1.3	1.3	1.0
Fiber, g	0.3	0.7	0.7	2.7
Vitamin E, mg	2.0	1.3	1.3	1.3
SFA/USFA	1∶3.5	1∶3.32	1∶3.44	1∶3.22
MUFA/PUFA	1∶2.06	1∶0.42	1∶0.46	1∶0.40
SFA/MUFA	1∶1.14	1∶2.33	1∶2.35	1∶2.48
SFA/PUFA	1∶2.35	1∶0.98	1∶1.09	1∶0.73

Abbreviations: Kcal: kilocalories; g: gram; n-6: omega 6 (linoleic acid); SFA: saturated fatty acids; USFA: unsaturated fatty acids; MUFA: monounsaturated fatty acids; PUFA: polyunsaturated fatty acids; Product A: cocoa cream; Product B: cocoa+hazelnut cream; Product C: cocoa+hazelnut+phytosterols cream; Product D (for the purpose of the present study termed LMN product): cocoa+hazelnut+phytosterols+soluble fiber cream.

a Nutrient composition calculated from data provided by the manufacturers and with USDA food composition tables.

The participants, clinical investigators and laboratory personnel were blinded with respect to the type of cream being consumed.

### Statistical analysis

Results are expressed as mean ± SD, baseline-adjusted least square means (95% confidence intervals; 95%CI), median (inter-quartile range; 25^th^ percentile, 75^th^ percentile) or frequencies and percentages (%).

We analyzed all continuous variables that had Gaussian distributions using standard ANCOVA (or mixed models when the endpoints were measured repeatedly over time) with the (co)variance type set to unstructured [14], including the baseline observation as a covariate in both cases. For the rest of continuous, but non-Gausian variables, we used the same model with a prior rank transformation of the dependent variable [15]. The same model was applied for ICAM and VCAM for additional and suportive analyses after a prior log10 transformation of the data. For measures other than the anthropometric and clinical variables, the comparisons versus control were all included in the tables, with further pair-wise comparisons included. Only comparisons versus control were conducted for the rest of variables. Fisher's protected Least Significance Difference (LSD) method was used to control for type I error and. As such, pair-wise treatment inferential comparisons were applied only if this factor was significant.

The main analyses were performed with the intent-to-treat (ITT) population i.e. subjects with at least one post-baseline measurement. A secondary analysis was applied to a per-protocol (PP) subset of patients who were free of any major violations of the protocol. The analyses shown in the text and tables were performed on the ITT population. The sensitivity analyses performed on the PP population gave very similar results.

The level of significance was set at the standard two-sided level of 5%. All analyses were performed using SAS (version 9.1.3) software (SAS Institute Inc., Cary, NC, USA).

## Results

### Participant flow

**Figure 1:**
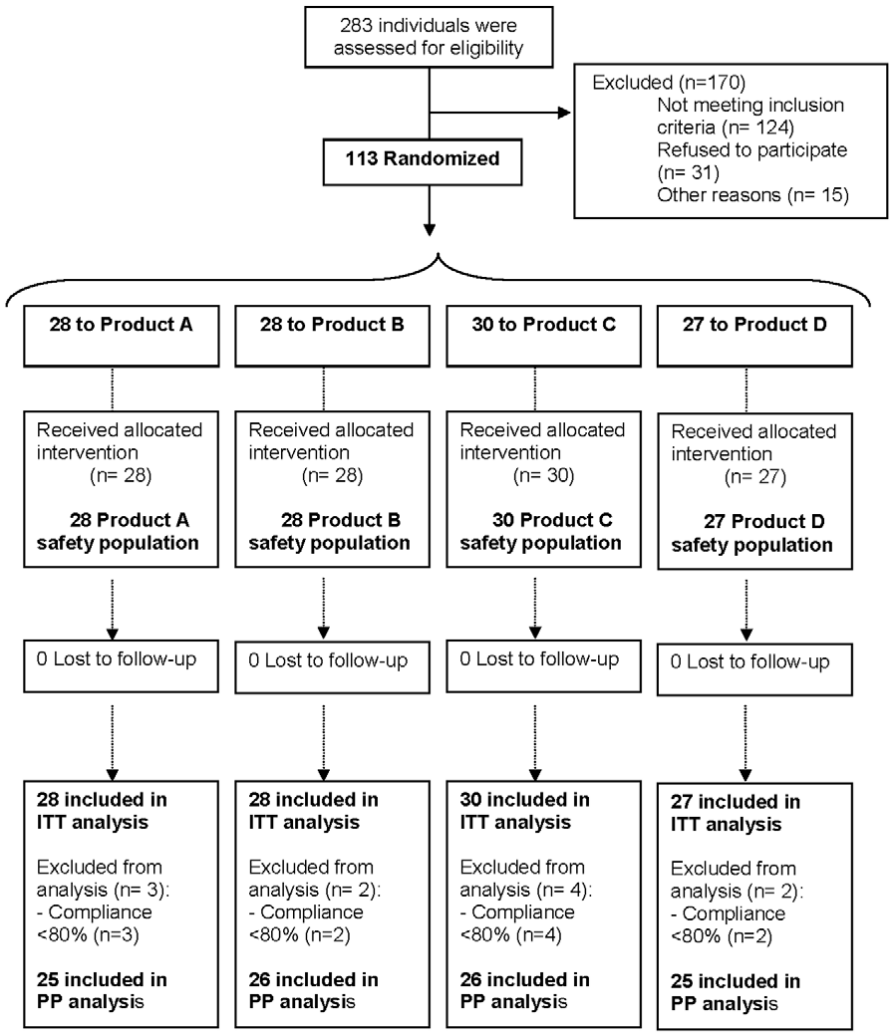
Flow diagram of participants. From the ITT population (n = 113), 11 participants were excluded because of lack of compliance with product consumption (i.e. <80% product consumption). Hence, the PP population was 102. Product A: cocoa-only cream; Product B: cocoa+hazelnut cream; Product C: cocoa+hazelnut+phytosterols cream; Product D (for the purpose of the present study termed LMN product): cocoa+hazelnut+phytosterols+soluble fiber cream; ITT: intention to treat; PP: per protocol.

Figure 1 describes the flow of participants through the study.

### Recruitment

The participants were recruited between April 2005 and December 2005.

### Baseline data; Characteristics of participants

The groups receiving the 4 different cocoa cream products appeared to have some differences at baseline; group A had the youngest individuals while group B contained fewer individuals with high BP and family history of premature CVD. No significant differences were observed in the other baseline characteristics (Table 2).

**Table 2:** Baseline characteristics of study participants.

	Product A (n = 28)	Product B (n = 28)	Product C (n = 30)	Product LMN (n = 27)	Total (n = 113)
Age, years	49.79±9.53	56.79±10.46	56.03±10.06	53.33±8.42	54.03±9.93
BMI, Kg/m^2^	28.31±3.25	27.30±3.01	28.53±3.22	28.28±3.56	28.12±3.25
Gender, male	11 (39.3%)	12 (42.9%)	13 (43.3%)	10 (37.0%)	46 (40.7%)
CVD risk factor:					
Age	16 (57.1%)	23 (82.1%)	22 (73.3%)	20 (74.1%)	81 (71.7%)
Smoker	8 (28.6%)	8 (28.6%)	7 (23.3%)	5 (18.5%)	28 (24.8%)
High BP	12 (42.9%)	8 (28.6%)	13 (43.3%)	11 (40.7%)	44 (38.9%)
HDL-c*	0 (0.0%)	1 (3.6%)	2 (6.7%)	4 (14.8%)	7 (6.2%)
F-HD^+^	6 (21.4%)	2 (7.1%)	3 (10.0%)	4 (14.8%)	15 (13.3%)

ITT population.

Results are expressed as means ± SD or frequencies (%).

Abbreviations: Product A: cocoa cream; Product B: cocoa+hazelnut cream; Product C: cocoa+hazelnut+phytosterols cream; Product D (for the purpose of the present study termed LMN): cocoa+hazelnut+phytosterols+soluble fiber cream; BMI: body mass index calculated as weight in kilograms divided by height in meters squared; CVD: cardiovascular disease; HDL-c: high density lipoprotein cholesterol, * <1.0 mmol/L (40 mg/dL) and <1.18 mmol/L (46 mg/dL) for men and women, respectively; F-HD: family history of premature heart disease, +in male <55 years, in female <65 years in first-degree relative.

### Numbers analyzed

There were 283 individuals who were considered eligible for the study (Figure 1). The 113 remaining participants were randomized in ITT analysis; 46 men and 67 women having completed the study. Finally, 11 participants were excluded from the PP analysis.

### Outcomes and estimation

The results (anthropometric measures, lipid profile, CVD biomarkers and dietary adherence) at 4 weeks of the intervention period are summarized in Tables S1, S2, S3 and S4.

The differences observed with respect to dietary fiber intake can be explained by the fiber content in the LMN product.

The consumption of the 4 cocoa products had similar effects on primary end-points in males and females.

Compliance was high: 94% with the 4 different cocoa cream products and 98% with the diet.

During the intervention period, the 4 groups consumed approximately 10% of energy as SFA (Table S4). Compared with the control group, inclusion of hazelnuts induced higher MUFA and lower PUFA content, while the product LMN group had a higher intake of dietary fiber (P<0.0001) (Table S4).

### Anthropometric and clinical measurements

No changes in bodyweight and WC measurements were observed at the end of the intervention period (Table S1). Surprisingly, 4 weeks on product A (designated as control) reduced systolic BP (−7.89 mmHg; 95%CI: −11.45 to −4.3) and diastolic BP (−5.54 mmHg; 95%CI: −7.79 to −3.29).

These intra-group changes for group A were greater than that observed in the other 3 treatment groups. The between-group comparisons showed no statistically significant differences *vs.* control in systolic BP, and only group B was significantly different from control with respect to diastolic BP (P = 0.0357).

The treatment effect was not heterogeneous between individuals who were pre-hypertensive and those at stage-1 hypertension.

#### Lipid profile

Compared to the control group, after 4 weeks product C reduced LDL-c by −11.2% (0.47 mmol/L; 95%CI: 0.23 to 0.71; P = 0.0002), and Apo B/Apo A ratio by −7.8% (0.054; 95%I: 0.014 to 0.094; P = 0.0085). LMN cream decreased LDL-c by 9.2% (0.39 mmol/L; 95%CI: 0.15 to 0.64; P = 0.0018) and Apo B/Apo A ratio by −10.5% (0.074; 95%CI: 0.033 to 0.115; P = 0.0005) (Table S2).

#### CVD biomarkers

Compared with control group, LMN reduced hsCRP by 0.96 mg/L (95%CI: 0.02 to 2.04; P<0.0083) and oxLDL by 4.0 U/L (95%CI: 0.51 to 7.51; P = 0.0252) (Table S3).

### Ancillary analyses

#### Endo-PAT subgroup

A subgroup of participants (n = 14) from within the control product group A were tested for endothelial function pre - and 2-week post-intervention using the Endo-PAT system. We observed that the consumption of cocoa improved the vasodilator response (as measured by fingertip tonometry) from 1.800 to 2.173 AU, pre- *versus* post-intervention (mean 0.373 AU; 95%CI: −0.044 to 0.702; P = 0.029).

### Adverse events

All participants consumed the cocoa cream during the stabilization period and none reported any adverse effects. During the intervention period, two participants reported a bloating feeling and one reported poor appetite.

## Discussion

### Interpretation

Cocoa cream containing other palatable ingredients such as those of the “portfolio” diet (in the current study termed the LMN product) beneficially modulates the CVD biomarkers measured [16]–[18].

Compared to control cocoa cream, the reductions in plasma LDL-c observed with cocoa+hazelnuts+phytosterols of −11.2% and with LMN of −9.2% are of similar magnitude to that of sterols and stanols (2 g/d) or of *Plantago ovata* husk (14 g/d) [19]. Of note is that the phytosterols+soluble fiber combination had a similar lowering effect on plasma LDL-c. However, contrary to expectation, the hypocholesterolemic effect was neither additive nor synergistic. Further, the cocoa+hazelnuts+phytosterols cream reduced the ApoB/ApoA ratio by about −11.8% and LMN reduced the ratio by −10.5% due, mainly, to a moderate reduction in plasma Apo B-100. Since Apo B is considered the better indicator of CVD risk than the conventional LDL-c status [20], [21], the reduction of the ApoB/ApoA ratio to values <0.7 becomes a therapeutic target [20]–[23].

Compared to the control cream, LMN reduced oxLDL by −6%. In this case, the addition of soluble fiber reduced oxLDL plasma concentrations by about −5.9%; a similar LDL antioxidant reduction observed with the consumption of 14 g/d for 8 weeks of the soluble fiber *Plantago ovata* husk [19]. As such, soluble fiber addition can exert a greater LDL antioxidant effect than cocoa alone [24], [25].

Compared with control cocoa cream product, LMN reduced hsCRP by −33.4%, which indicates an anti-inflammatory effect of this product. Soluble fiber is the additional ingredient in the LMN product which may be responsible for this lowering of hsCRP; an effect which was recently described with high MUFA content in the portfolio diet [26] and the anti-inflammatory effect of cocoa polyphenols [27].

The large blood pressure lowering effect (systolic BP by −7.8 mm Hg and diastolic BP by −5.4 mm Hg) of the cocoa product warrants further investigation. Further studies on product A composition are underway to seek an explanation for the clinically significant reduction in BP we observed. Because of these reductions induced by the “control” product, no further statistical comparison *versus* the other manufactured products were made with respect to BP.

While the 4 cream products contained the same amount of cocoa and polyphenols i.e. about 1 g/unit together with 10 mg/unit of phenolic compounds and 10% of SFA, the addition of hazelnuts (30 g/) in the 3 other cream products changed the MUFA contribution to 24% and PUFA to about 10%. This produces a dietary composition of 20% MUFA, 5% PUFA and 10% SFA; a composition that is recommended by scientific societies [7], [8]. The sugar content of the 4 creams accounts for ≤50% of total energy. In the present study, participants maintained their weight and WC throughout the intervention period, reinforcing the benefits of the cocoa intake traded-off against sugar, fat and calorie count [28]. In a subgroup of participants consuming the cocoa cream product A, an arterial vasodilatory effect was observed, as measured by Endo-PAT which provides a quantitative evaluation of endothelial dysfunction [29]. Recently, the digital Endo-PAT device was reported not only to predict patients with ischemic heart disease [29] but also an improvement in endothelial function as a consequence of cocoa product consumption [30]. The arterial vasodilatory effect of cocoa cream observed in present study could explain, at least in part, the reduction in BP.

### Generalizability

The present exploratory study showed that cocoa extracts, mixed with other food ingredients (such as those in the “portfolio diet”) to produce a palatable cream product is a new alternative to improve CVD biomarkers, despite the consumption being over a very short time in the present trial. These CVD beneficial effects need to be demonstrated over a longer-term of consumption, and with doses which can be incorporated within a diet recommendable for CVD risk reduction.

### Limitations

The study period with cocoa cream products focused on short-term surrogate markers of inflammation, as opposed to clinically relevant outcomes in the patients. The optimum daily intake of the products to maximize the benefit remains to be determined. An appropriate control product is needed since even the cocoa cream alone (product A), although effective as control for LDL-c plasma level outcomes, had a BP lowering effect.

### Overall evidence

We conclude that palatable cocoa cream products, particularly the LMN cream, have anti-inflammatory and antioxidant effects in addition to a beneficial lowering effect on LDL-c, Apo B and ApoB/ApoA ratio. Thus, the effect of soluble fiber amplified with sterols (as contained in the cocoa creams) provides new dietary therapeutic perspectives in addressing cardiovascular disease.

## Supporting Information

Table S1 Anthropometric and clinical measures. ITT population.
(DOC)

Table S2 Lipid profile variables. ITT population.
(DOC)

Table S3 Oxidative stress, endothelial dysfunction, inflammation, anti-thrombotic activity and syndrome biomarkers. ITT population.
(DOC)

Table S4 Dietary composition for each group after 4 weeks of treatment. ITT population.
(DOC)

Checklist S1 **CONSORT checklist.**
(DOC)

Protocol S1 **Trial protocol.**
(DOC)
