# Supplementary material for: Cocoa, Hazelnuts, Sterols and Soluble Fiber Cream Reduces Lipids and Inflammation Biomarkers in Hypertensive Patients: A Randomized Controlled Trial
Source: PLoS One. 2012 Feb 27;7(2):e31103. doi: 10.1371/journal.pone.0031103 (PMC3287993; doi:10.1371/journal.pone.0031103)
Supplement: Table S1 — Anthropometric and clinical measures. ITT population. (DOC) [file pone.0031103.s001.doc]

**Table S1.** Anthropometric and clinical measures. ITT population

| Variables | Product | Baseline  Mean±SD | Baseline change at 4 weeks  Adjusted Mean [95%CI]  (*% change from baseline*)* | | Adjusted Mean [95%CI]  (*% difference from control*) | | *P-value* *control vs. product *** | *Overall*  *P-value **** |
| --- | --- | --- | --- | --- | --- | --- | --- | --- |
| Weight, Kg | A | 77.32±10.83 | 0.236  [-0.275 to 0.748] | *(0.3%)* |  |  |  | 0.403 |
|  | B | 72.13±10.88 | 0.008  [-0.510 to 0.527] | *(0.0%)* | 0.228 [-0.505 to 0.962] | *(-0.3%)* | 0.539 |  |
|  | C | 73.55±10.55 | -0.004  [-0.483 to 0.474] | *(0.0%)* | 0.241 [-0.461 to 0.943] | *(-0.3%)* | 0.497 |  |
|  | LMN | 73.43±12.23 | -0.401 [-0.941 to 0.138] | *(-0.5%)* | 0.638  [-0.108 to 1.383] | *(-0.9%)* | 0.093 |  |
| BMI, Kg/m^2^ | A | 28.31±3.255 | 0.082  [-0.095 to 0.260] | *(0.3%)* |  |  |  | 0.360 |
|  | B | 27.30±3.016 | -0.022 [-0.205 to 0.160] | *(-0.1%)* | 0.104  [-0.151 to 0.360] | *(-0.4%)* | 0.418 |  |
|  | C | 28.53±3.223 | 0.000  [-0.168 to 0.168] | *(0.0%)* | 0.082 [-0.162 to 0.327] | *(-0.3%)* | 0.506 |  |
|  | LMN | 28.28±3.568 | -0.151 [-0.341 to 0.038] | *(-0.5%)* | 0.233 [-0.026 to 0.493] | *(-0.8%)* | 0.078 |  |
| WC, cm | A | 95.52±8.138 | -0.579 [-1.776 to 0.617] | *(-0.6%)* |  |  |  | 0.401 |
|  | B | 93.66±7.871 | -1.899 [-3.096 to -0.702] | *(-2.0%)* | 1.320 [-0.374 to 3.015] | *(-1.4%)* | 0.126 |  |
|  | C | 95.68±8.867 | -1.737 [-2.915 to -0.560] | *(-1.9%)* | 1.158 [-0.517 to 2.833] | *(-1.2%)* | 0.173 |  |
|  | LMN | 93.18±9.720 | -1.647 [-2.868 to -0.427] | *(-1.8%)* | 1.068  [-0.644 to 2.782] | *(-1.2%)* | 0.219 |  |
| SBP, mm Hg | A | 124.7±12.65 | -7.894 [-11.450 to -4.338] |  |  |  |  | 0.234 |
|  | B | 127.4±13.07 | -2.982  [-6.499 to 0.533] |  | -4.911 [-9.888 to 0.065] |  | 0.053 |  |
|  | C | 131.9±16.32 | -4.429  [-7.840 to -1.019] |  | -3.464 [-8.433 to 1.503] |  | 0.170 |  |
|  | LMN | 132.3±12.24 | -3.871 [-7.470 to -0.273] |  | -4.022 [-9.128 to 1.083] |  | 0.121 |  |
| DBP, mm Hg | A | 84.86±7.847 | -5.544 [-7.794 to -3.294] |  |  |  |  | 0.083 |
|  | B | 83.38±8.619 | -2.157  [-4.420 to 0.106] |  | -3.387 [-6.575 to -0.199] |  | 0.038 |  |
|  | C | 83.87±7.570 | -3.126 [-5.307 to -0.945] |  | -2.418  [-5.549 to 0.713] |  | 0.129 |  |
|  | LMN | 88.85±8.808 | -1.650  [-3.997 to 0.696] |  | -3.894 [-7.153 to -0.636] |  | 0.020 |  |

Results are expressed as means ± SD and baseline adjusted least square means [95%CI].

*Mean mm Hg change = ([Mean baseline] – [Adjusted Mean at week 4] / [Mean baseline]).

Abbreviations: Product A: cocoa cream considered as control; Product B: cocoa + hazelnut cream; Product C: cocoa + hazelnut + phytosterols cream; Product D: (for the purpose of the present study termed LMN): cocoa + hazelnut + phytosterols + soluble fiber cream; BMI: body mass index calculated as weight in kilograms divided by height in meters squared; WC: waist circumference; SBP: systolic blood pressure; DBP: diastolic blood pressure. ITT: intent-to-treat.

** P values indicate control *vs.* product comparison of baseline-adjusted mean differences

*** P values indicate the overall significance for the treatment group effect. P-values for the control vs product comparisons should only be considered for inferential purposes when the overall P-value is statistically significant at the 5% level.
