# Supplementary material for: Cocoa, Hazelnuts, Sterols and Soluble Fiber Cream Reduces Lipids and Inflammation Biomarkers in Hypertensive Patients: A Randomized Controlled Trial
Source: PLoS One. 2012 Feb 27;7(2):e31103. doi: 10.1371/journal.pone.0031103 (PMC3287993; doi:10.1371/journal.pone.0031103)
Supplement: Table S2 — Lipid profile variables. ITT population. (DOC) [file pone.0031103.s002.doc]

**Table S2**. Lipid profile variables. ITT population

| Variables | Product | Baseline  Mean±SD | Baseline change at 4 weeks  Adjusted Mean [95%CI]  (*% change from baseline*)* | | Adjusted Mean [95%CI]  (*% difference from control* ) | | *P-value control vs. product*** | Other significant  *P* | *Overall*  *P-value **** |
| --- | --- | --- | --- | --- | --- | --- | --- | --- | --- |
| TC, mmol/L | A | 6.141±0.860 | -0.087 [-0.242 to 0.066] | *(-1.4%)* |  |  |  |  | <0.001 |
|  | B | 6.098±0.801 | 0.0075  [-0.146 to 0.161] | *(0.1%)* | -0.095 [-0.313 to 0.123] | *(1.6%)* | 0.389 | C, LMN |  |
|  | C | 6.005±0.809 | -0.5412 [-0.690 to -0.391] | *(-8.9%)* | 0.4534  [0.238 to 0.668] | *(-7.4%)* | 0.001 |  |  |
|  | LMN | 6.189±0.692 | -0.501 [-0.658 to -0.343] | *(-8.1%)* | 0.413  [0.192 to 0.633] | *(-6.7%)* | 0.003 |  |  |
| LDL-c, mmol/L | A | 4.244±0.673 | -0.002 [-0.173 to 0.167] | *(-0.1%)* |  |  |  |  | <0.001 |
|  | B | 4.195±0.720 | 0.068  [-0.102 to 0.239] | *(1.7%)* | -0.071  [-0.312 to 0.170] | *(1.7%)* | 0.559 | C, LMN |  |
|  | C | 4.150±0.911 | -0.471 [-0.636 to -0.306] | *(-11.2%)* | 0.468  [0.231 to 0.706] | *(-11.2%)* | 0.002 |  |  |
|  | LMN | 4.278±0.557 | -0.396  [-0.570 to -0.222] | *(-9.3%)* | 0.393  [0.149 to 0.636] | *(-9.2%)* | 0.002 |  |  |
| HDL-c, mmol/L | A | 1.219±0.305 | -0.019  [-0.077 to 0.039] | *(-1.6%)* |  |  |  |  | 0.808 |
|  | B | 1.254±0.323 | -0.048  [-0.106 to 0.010] | *(-3.9%)* | 0.028  [-0.053 to 0.111] | *(-2.3%)* | 0.4928 |  |  |
|  | C | 1.224±0.355 | -0.0514 [-0.107 to 0.004] | *(-4.1%)* | 0.0321  [-0.048 to 0.113] | *(-2.5%)* | 0.434 |  |  |
|  | LMN | 1.264±0.419 | -0.0231 [-0.082 to 0.036] | *(-1.8%)* | 0.0038  [-0.079 to 0.087] | *(-0.2%)* | 0.929 |  |  |
| TG, mmol/L | A | 1.419±0.894 | -0.102  [-0.320 to 0.114] | *(-7.2%)* |  |  |  |  | 0.875 |
|  | B | 1.374±0.958 | -0.036  [-0.253 to 0.181] | *(-2.8%)* | -0.066 [-0.374 to 0.240] | *(4.4%)* | 0.668 |  |  |
|  | C | 1.288±0.892 | -0.031  [-0.241 to 0.178] | *(-2.3%)* | -0.071 [-0.374 to 0.231] | *(5.0%)* | 0.641 |  |  |
|  | LMN | 1.327±0.920 | -0.139  [-0.360 to 0.08] | *(-10.5%)* | 0.036 [-0.273 to 0.347] | *(-3.3%)* | 0.816 |  |  |
| Apo B-100, g/L | A | 1.158±0.192 | 0.034  [-0.006 to 0.076] | *(3.0%)* |  |  |  |  | 0.001 |
|  | B | 1.161±0.160 | 0.039  [-0.001 to 0.079] | *(3.5%)* | -0.004 [-0.062 to 0.053] | *(0.5%)* | 0.873 | C, LMN |  |
|  | C | 1.116±0.202 | -0.059  [-0.098 to -0.019] | *(-5.1%)* | 0.093  [0.036 to 0.150] | *(-8.1%)* | 0.002 |  |  |
|  | LMN | 1.171±0.193 | -0.064  [-0.105 to -0.022] | *(-5.5%)* | 0.098  [0.04 to 0.157] | *(-8.5%)* | 0.001 |  |  |
| Apo A-1, g/L | A | 1.676±0.322 | 0.0089  [-0.040 to 0.058] | *(0.5%)* |  |  |  |  | 0.856 |
|  | B | 1.742±0.252 | 0.022  [-0.026 to 0.070] | *(1.3%)* | -0.013 [-0.082 to 0.056] | *(0.8%)* | 0.707 |  |  |
|  | C | 1.661±0.295 | 0.010  [-0.036 to 0.057] | *(0.6%)* | -0.001 [-0.069 to 0.066] | *(0.1%)* | 0.966 |  |  |
|  | LMN | 1.717±0.243 | 0.036  [-0.013 to 0.085] | *(2.1%)* | -0.027 [-0.097 to 0.0431] | *(1.6%)* | 0.445 |  |  |
| Apo B/ApoA ratio, (1/1) | A | 0.710±0.153 | 0.020  [-0.008 to 0.049] | *(2.9%)* |  |  |  |  | 0.007 |
|  | B | 0.683±0.155 | 0.011  [-0.016 to 0.040] | *(1.7%)* | 0.008 [-0.032 to 0.048] | *(-1.2%)* | 0.683 | C, LMN |  |
|  | C | 0.698±0.205 | -0.033 [-0.060 to -0.006] | *(-4.9%)* | 0.053  [0.014 to 0.093] | *(-7.8%)* | 0.009 |  |  |
|  | LMN | 0.704±0.187 | -0.053 [-0.082 to -0.024] | *(-7.6%)* | 0.074  [0.033 to 0.114] | *(-10.5%)* | 0.001 |  |  |

Results are expressed as means ± SD and baseline adjusted least square means [95%CI].

* Mean relative change = ([Mean baseline] – [Adjusted Mean at week 4]) / [Mean baseline]

Abbreviations: Product A: cocoa cream considered as control; Product B: cocoa + hazelnut cream; Product C: cocoa + hazelnut + phytosterols cream; Product D: (for the purpose of the present study termed LMN): cocoa + hazelnut + phytosterols + soluble fiber cream; TC: total cholesterol; LDL-c: low density lipoprotein cholesterol; HDL-c: high density lipoprotein cholesterol; TG: triglycerides; Apo B-100: apolipoprotein B; Apo A-1: apolipoprotein A. ITT: intent-to-treat.

To convert international units (SI) to conventional units (mg/dL): For cholesterol multiply by 38.7; For TG multiply by 88.5; For Apo A-1 and Apo B-100 multiply by 100.

** P values indicate control *vs*. product comparison of baseline-adjusted mean differences

*** P values indicate the overall significance for the treatment group effect. P-values for pairwise treatment group comparisons should only be considered for inferential purposes when the overall P-value is statistically significant at the 5% level.
