# Supplementary material for: Cocoa, Hazelnuts, Sterols and Soluble Fiber Cream Reduces Lipids and Inflammation Biomarkers in Hypertensive Patients: A Randomized Controlled Trial
Source: PLoS One. 2012 Feb 27;7(2):e31103. doi: 10.1371/journal.pone.0031103 (PMC3287993; doi:10.1371/journal.pone.0031103)
Supplement: Table S3 — Oxidative stress, endothelial dysfunction, inflammation, anti-thrombotic activity and syndrome biomarkers. ITT population. (DOC) [file pone.0031103.s003.doc]

**Table S3**. Oxidative stress, endothelial dysfunction, inflammation, anti-thrombotic activity and syndrome biomarkers. ITT population

| Variables | Product | Baseline  Mean±SD | Baseline change at 4 weeks  Adjusted Mean [95%CI]  (*% change from baseline*)* | | Adjusted Mean [95%CI]  (*% difference from control* ) | | *P-value control vs. product*** | Other significant  *P* | *Overall*  *P-value **** |
| --- | --- | --- | --- | --- | --- | --- | --- | --- | --- |
| oxLDL, mU/L | A | 70.32±15.30 | -2.092  [-4.555 to 0.370] | *(-3.0%)* |  |  |  |  | 0.026 |
|  | B | 65.10±10.08 | -1.122  [-3.585 to 1.340] | *(-1.7%)* | -0.970 [-4.470 to 2.529] | *(1.3%)* | 0.584 | LMN |  |
|  | C | 66.75±12.51 | -4.265  [-6.635 to -1.896] | *(-6.6%)* | 2.173  [-1.250 to 5.597] | *(-3.6%)* | 0.211 |  |  |
|  | LMN | 68.75±15.44 | -6.101  [-8.599 to -3.603] | *(-8.9%)* | 4.0084  [0.507 to 7.509] | *(-5.9%)* | 0.025 |  |  |
| VCAM-1, ng/mL | A | 550.7±178.6 | -16.353 [-53.520 to 20.813] | *(-3.0%)* |  |  |  |  | 0.952 |
|  | B | 640.8±396.8 | -14.895 [-51.973 to 22.181] | *(-2.4%)* | -1.4575 [-54.148 to 51.233] | *(0.5%)* | 0.956 |  |  |
|  | C | 614.5±191.4 | -21.146 [-56.880 to 14.587] | *(-3.3%)* | 4.793 [-46.825 to 56.411] | *(-0.3%)* | 0.854 |  |  |
|  | LMN | 605.2±224.9 | -6.074 [-43.731 to 31.583] | *(-1.0%)* | -10.2792 [-63.200 to 42.641] | *(2.0%)* | 0.701 |  |  |
| Log_10_ transformed VCAM-1, ng/mL | A | 2.722±0.126 | 1.582 [1.359 to 1.805] | *(7.3%)* |  |  |  |  | 0.003 |
|  | B | 2.762±0.180 | 1.926 [1.664 to 2.189] | *(14.6%)* | -0.3443 [-0.691 to 0.002] | *(7.4%)* | 0.052 |  |  |
|  | C | 2.468±0.135 | 1.439 [1.243 to 1.635] | *(4.7%)* | 0.1431  [-0.155 to 0.441] | *(-2.6%)* | 0.339 | B, LMN |  |
|  | LMN | 2.758±0.142 | 1.984 [1.722 to 2.246] | *(16.8%)* | -0.4017 [-0.745 to 0.058] | *(9.6%)* | 0.023 |  |  |
| ICAM-1, ng/mL | A | 316.0±107.4 | -0.347 [-24.737 to 24.042] | *(-0.1%)* |  |  |  |  | 0.994 |
|  | B | 318.8±115.3 | -2.689 [-27.069 to 21.689] | *(-0.8%)* | 2.3425 [-32.120 to 36.805] | *(-0.7%)* | 0.893 |  |  |
|  | C | 343.0±143.5 | -3.994  [-27.598 to 19.610] | *(-1.3%)* | 3.6466  [-30.346 to 37.639] | *(-1.1%)* | 0.832 |  |  |
|  | LMN | 325.5±115.9 | -5.079 [-29.8942 to 19.7361] | *(-1.6%)* | 4.731 [-30.061 to 39.524] | *(-1.5%)* | 0.788 |  |  |
| Log_10_ transformed ICAM-1, ng/mL | A | 2.477±0.145 | 1.4722 [1.2198 to 1.7247] | *(9.9%)* |  |  |  |  | 0.268 |
|  | B | 2.475±0.164 | 1.7112 [1.4335 to 1.9889] | *(17.2%)* | -0.239 [-0.616 to 0.138] | *(7.4%)* | 0.736 |  |  |
|  | C | 2.499±0.183 | 1.3608 [1.1224 to 1.5992] | *(7.3%)* | 0.111 [-0.234 to 0.456] | *(-2.6%)* | 0.209 |  |  |
|  | LMN | 2.480±0.187 | 1.4128 [1.1688 to 1.6568] | *(8.6%)* | 0.059 [-0.292 to 0.411] | *(-1.3%)* | 0.064 |  |  |
| IL-6, pm/mL | A | 0.301 (0.168 to 0.484) | -0.017  (-0.0102 to 0.037) | *(-5.6%)* |  |  |  |  | 0.261 |
|  | B | 0.296 (0.214 to 0.398) | 0.035  (-0.068 to 0.148) | *(11.8%)* | -0.067 (-0.262 to 0.090) | *(17.5%)* | 0.1417 |  |  |
|  | C | 0.290 (0.180 to 0.480) | 0.023  (-0.050 to 0.239) | *(7.9%)* | -0.080 (-0.290 to 0.060) | *(13.6%)* | 0.0601 |  |  |
|  | LMN | 0.265 (0.140 to 0.416) | 0.024  (-0.130 to 0.152) | *(9.1%)* | -0.052 (-0.229 to 0.139) | *(14.7%)* | 0.387 |  |  |
| hsCRP, mg/L | A | 1.635 (0.765 to 3.240) | 0.115  (-0.435 to 0.900) | *(7.0%)* |  |  |  |  | 0.054 |
|  | B | 2.780 (1.280 to 5.300) | -0.190  (-1.080 to 0.115) | *(-6.8%)* | 0.590  (-0.475 to 1.640) | *(-13.9%)* | 0.059 |  |  |
|  | C | 2.130 (1.380 to 3.020) | -0.025  (-0.460 to 0.280) | *(-1.2%)* | 0.375 (-0.415 to 1.220) | *(-8.2%)* | 0.244 |  |  |
|  | LMN | 2.120 (1.250 to 5.190) | -0.560  (-1.80 to -0.030) | *(-26.4%)* | 0.955 (0.020 to 2.035) | *(-33.4%)* | 0.008 |  |  |

Results are expressed as means ± SD and baseline adjusted least square means [95%CI] for all parameters except IL-6 and hsCRP. For the latter variables the analyses were non-parametric and the estimators were median (25^th^ percentile – 75^th^ percentile).

* Mean relative change = ([Mean baseline] – [Adjusted Mean at week 4]) / [Mean baseline]. Values are log10 transformed before applying this formula.

Abbreviations: Product A: cocoa cream considered as control; Product B: cocoa + hazelnut cream; Product C: cocoa + hazelnut + phytosterols cream; Product D (for the purpose of the present study termed LMN): cocoa + hazelnut + phytosterols + soluble fiber cream; oxLDL: oxidized LDL; VCAM-1: vascular adhesion molecule-1; ICAM-1: intercellular adhesion molecule-1; IL-6: interleukin-6; hsCRP: high sensitivity C-reactive protein. ITT: intent-to-treat.

** P values indicate control vs. product comparison of baseline-adjusted mean differences

*** P values indicate the overall significance for the treatment group effect. P-values for the control vs product comparisons should only be considered for inferential purposes when the overall P-value is statistically significant at the 5% level.
