# Supplementary material for: Cocoa, Hazelnuts, Sterols and Soluble Fiber Cream Reduces Lipids and Inflammation Biomarkers in Hypertensive Patients: A Randomized Controlled Trial
Source: PLoS One. 2012 Feb 27;7(2):e31103. doi: 10.1371/journal.pone.0031103 (PMC3287993; doi:10.1371/journal.pone.0031103)
Supplement: Table S4 — Dietary composition for each group after 4 weeks of treatment. ITT population. (DOC) [file pone.0031103.s004.doc]

**Table S4**. Dietary composition for each group after 4 weeks of treatment. ITT population

| Variables | Product | Baseline  Mean±SD | Baseline change at 4 weeks  Adjusted Mean [95%CI]  (*% change from baseline*)* | | Adjusted Mean [95%CI]  (*% difference from control* ) | | *P-value*  *control vs.*  *product *** | Other significant  *P-values* | *Overall*  *P-value **** |
| --- | --- | --- | --- | --- | --- | --- | --- | --- | --- |
| Energy, Kcal/d | A | 2347±514.3 | -238.4722 [-368.810 to -108.134] | *(-10.2%)* |  |  |  |  | 0.429 |
|  | B | 2408±659.9 | -344.4643 [-474.807 to -214.121] | *(-14.5%)* | 105.992 [-78.4079 to 290.392] | *(-4.3%)* | 0.257 |  |  |
|  | C | 2381±549.1 | -230.0918 [-355.965 to -104.218] | *(-9.6%)* | -8.3804 [-189.5851 to 172.8243] | *(0.6%)* | 0.927 |  |  |
|  | LMN | 2371±477.0 | -342.0286 [-474.711 to -209.345] | *(-14.4%)* | 103.5564 [-82.4222 to 289.535] | *(-4.3%)* | 0.272 |  |  |
| CHO, % of total energy | A | 37.60±8.738 | 3.150  [0.803 to 5.498] | *(8.4%)* |  |  |  |  | 0.651 |
|  | B | 38.45±6.559 | 1.235  [-1.093 to 3.563] | *(3.2%)* | 1.915  [-1.365 to 5.196] | *(-5.2%)* | 0.220 |  |  |
|  | C | 41.86±4.656 | 2.691  [0.419 to 4.963] | *(6.4%)* | 0.459  [-2.852 to 3.77] | *(-1.9%)* | 0.784 |  |  |
|  | LMN | 40.73±5.702 | 2.941  [0.574 to 5.308] | *(7.2%)* | 0.209  [-3.145 to 3.564] | *(-1.2%)* | 0.902 |  |  |
| Protein, % of total energy | A | 14.70±2.538 | 0.324  [-0.422 to 1.07] | *(2.2%)* |  |  |  |  | 0.135 |
|  | B | 13.99±1.881 | 1.107  [0.366 to 1.848] | *(7.9%)* | -0.783  [-1.837 to 0.271] | *(5.7%)* | 0.144 |  |  |
|  | C | 13.67±1.985 | 1.493  [0.774 to 2.213] | *(10.9%)* | -1.169  [-2.212 to -0.126] | *(8.7%)* | 0.028 |  |  |
|  | LMN | 14.20±2.855 | 1.324  [0.57 to 2.079] | *(9.3%)* | -1.00  [-2.06 to 0.06] | *(7.1%)* | 0.064 |  |  |
| Total fat, % of total energy | A | 44.17±4.938 | -3.528  [-5.642 to -1.415] | *(-8.0%)* |  |  |  |  | 0.878 |
|  | B | 45.55±4.801 | -3.469  [-5.587 to -1.351] | *(-7.6%)* | -0.059  [-3.056 to 2.937] | *(0.4%)* | 0.969 |  |  |
|  | C | 43.30±4.447 | -4.376  [-6.431 to -2.322] | *(-10.1%)* | 0.848  [-2.092 to 3.788] | *(-2.1%)* | 0.569 |  |  |
|  | LMN | 45.72±8.581 | -4.369  [-6.528 to -2.21] | *(-9.6%)* | 0.841  [-2.186 to 3.868] | *(-1.6%)* | 0.583 |  |  |
| SFA, % of total energy | A | 12.65±1.891 | -3.025  [-3.926 to -2.124] | *(-23.9%)* |  |  |  |  | 0.941 |
|  | B | 13.22±2.343 | -3.042  [-3.943 to -2.14] | *(-23.0%)* | 0.017  [-1.26 to 1.294] | *(0.9%)* | 0.980 |  |  |
|  | C | 12.86±2.113 | -3.056  [-3.926 to -2.186] | *(-23.8%)* | 0.031  [-1.221 to 1.283] | *(0.1%)* | 0.961 |  |  |
|  | LMN | 12.98±3.431 | -2.708  [-3.625 to -1.792] | *(-20.9%)* | -0.317  [-1.603 to 0.969] | *(3.1%)* | 0.627 |  |  |
| MUFA, % of total energy | A | 20.13±4.005 | -0.82  [-2.063 to 0.422] | *(-4.1%)* |  |  |  |  | 0.058 |
|  | B | 20.86±2.294 | 1.583  [0.341 to 2.826] | *(7.6%)* | -2.404  [-4.163 to -0.644] | *(11.7%)* | 0.008 |  |  |
|  | C | 19.47±2.538 | -0.086  [-1.299 to 1.127] | *(-0.4%)* | -0.734  [-2.463 to 0.995] | *(3.6%)* | 0.402 |  |  |
|  | LMN | 21.57±4.460 | 0.343  [-0.936 to 1.622] | *(1.6%)* | -1.163  [-2.954 to 0.627] | *(5.7%)* | 0.202 |  |  |
| PUFA, % of total energy | A | 7.327±1.005 | 0.426  [0.032 to 0.819] | *(5.8%)* |  |  |  |  | <0.001 |
|  | B | 7.654±1.352 | -1.835  [-2.229 to -1.441] | *(-24.0%)* | 2.261  [1.703 to 2.818] | *(-29.8%)* | <0.001 |  |  |
|  | C | 7.121±1.638 | -2.161  [-2.544 to -1.778] | *(-30.3%)* | 2.587  [2.04 to 3.134] | *(-36.2%)* | <0.001 |  |  |
|  | LMN | 7.796±1.107 | -1.911  [-2.314 to -1.507] | *(-24.5%)* | 2.336  [1.771 to 2.901] | *(-30.3%)* | <0.001 |  |  |
| Dietary fiber, g/d | A | 21.93±5.934 | 0.1967  [-2.386 to 2.779] | *(0.9%)* |  |  |  |  | <0.001 |
|  | B | 21.84±8.112 | 2.1777  [-0.4054 to 4.760] | *(9.7%)* | -1.981 [-5.6329 to 1.6709] | *(8.8%)* | 0.285 | LMN |  |
|  | C | 22.47±8.829 | 2.8735  [0.3783 to 5.368] | *(13.2%)* | -2.6769 [-6.2687 to 0.9149] | *(12.3%)* | 0.143 | LMN |  |
|  | LMN | 22.53±6.773 | 7.7223  [5.092 to 10.352] | *(34.3%)* | -7.5257 [-11.2127 to -3.8386] | *(33.4%)* | <0.001 |  |  |

Results are expressed as means ± SD and baseline adjusted least square means [95%CI]. * Mean relative change = ([Mean baseline] – [Adjusted Mean at week 4]) / [Mean baseline]. Abbreviations: Product A: cocoa cream considered as control; Product B: cocoa + hazelnut cream; Product C: cocoa + hazelnut + phytosterols cream; Product D (for the purpose of the present study termed LMN): cocoa + hazelnut +phytosterols + soluble fiber cream; Kcal: kilocalories; CHO: carbohydrates; SFA: saturated fatty acids; MUFA: monounsaturated fatty acids; PUFA: polyunsaturated fatty acids. ITT: intent-to-treat. ** P values indicate control *vs.* product comparison of baseline-adjusted mean difference. *** P values indicate the overall significance for the treatment group effect. Pair-wise P-values should only be considered for inferential purposes when the overall P-value is statistically significant at the 5% level.
