# Supplementary material for: Cocoa, Hazelnuts, Sterols and Soluble Fiber Cream Reduces Lipids and Inflammation Biomarkers in Hypertensive Patients: A Randomized Controlled Trial
Source: PLoS One. 2012 Feb 27;7(2):e31103. doi: 10.1371/journal.pone.0031103 (PMC3287993; doi:10.1371/journal.pone.0031103)
Supplement: Protocol S1 — Trial protocol. (DOC) [file pone.0031103.s006.doc]

**MINISTERIO DE EDUCACIÓN Y CIENCIA**

**[Ministry of Education and Science]**

**DIRECCIÓN GENERAL DE INVESTIGACIÓN**

**[National Research Board]**

**Convocatoria de ayudas de Proyectos de Investigación**

**[*Convocation of Awards for Research Projects*]**

**MEMORIA CIENTÍFICO-TÉCNICA DEL PROYECTO**

**[Scientific-Technical Background of the Project]**

**SUMMARY/RESUMEN DE LA PROPUESTA**

**[*Summary of the Proposal*]**

**PI/INVESTIGADOR PRINCIPAL: Rosa SOLÀ ALBERICH**

**[Principal Investigator: Rosa Solà**

**PROJECT TITLE: Effects of cocoa cream products on cardiovascular disease risk factors**

| SUMMARY  (brief and precise, outlining only the most relevant topics and the proposed objectives): |
| --- |
| Dietary factors influence plasma lipid levels (such as low-density lipoprotein cholesterol; LDL-c), blood pressure (BP), and other cardiovascular disease (CVD) biomarkers. Modification of nutritional components, consumption of specific foods, food additives and supplements are the major dietary approaches in ameliorating these effects.  The most beneficial CVD changes result from: reducing intake of saturated (SFA) and trans fats; adequate intake of polyunsaturated (PUFA) and increasing the amount of monounsaturated fats (MUFA); fortifying foods with plant stanols or sterols; adding nuts to the diet; increasing the intake of soluble fiber and soy protein; increasing the consumption of oily or fish-derived omega-3 fatty acid or cocoa products and flavonols [5,6]. However, the effects of integrating groups of these ingredients within a dietary product are unknown.  The aim of our study is to assess the effects of cocoa, or cocoa + hazelnuts, or cocoa + hazelnuts + phytosterols, or cocoa + hazelnuts + phytosterols + soluble fiber within a cream product that can be added to a calorie-balanced weight-maintaining diet on intermediate metabolic markers of CVD risk in pre-hypertensive and stage-1 hypertensive and hypercholesterolemic volunteers.  In this multi-centered, randomized, controlled, double-blinded, parallel trial, the volunteers (pre-hypertensive and stage-1 hypertensive and hypercholesterolemic individuals) receive one of 4 cocoa cream products (13g/unit; 1 g cocoa/unit, 6 units/d; 465 Kcal/d) added to a low-saturated fat diet for 4 weeks. The 4 groups are: A) cocoa cream alone, designated as control; B) cocoa + hazelnut cream (30g/d hazelnuts); C) cocoa + hazelnuts + phytosterols (2 g/d); and D) (n=27), cocoa + hazelnuts + phytosterols + soluble fiber (20g/d; designated “LMN product”). Primary outcome measures were BP, LDL-c, apolipoprotein B-100 (Apo B), ApoB/ApoA ratio, oxidized LDL (oxLDL) and high-sensitive C-reactive protein (hsCRP). ANCOVA statistical analyses are applied to all variables. Significance is set at p<0.05.  If any of the products tested show beneficial effects on CVD biomarkers, cocoa creams would provide new therapeutic options within a cardio-protective diet. |

**TITULO DEL PROYECTO**: **Efecto de varios bombones sobre factores de riesgo cardiovascular.**

| RESUMEN (debe ser breve y preciso, exponiendo sólo los aspectos más relevantes y los objetivos propuestos): |
| --- |
| Estudios llevados a cabo durante las últimas décadas han demostrado de forma concluyente que alimentos como los frutos secos, productos como el cacao o ingredientes como los fitosteroles, la fibra o el ácido fólico, al ser consumidos de forma aislada o al tomar varios de ellos integrados en una misma dieta pueden contribuir a la prevención o al tratamiento de las enfermedades cardiovasculares.  El objetivo global del proyecto es evaluar si al optimizar la composición de bombones que incluyan ingredientes naturales con demostrada actividad biológica se observan efectos cardioprotectores en el hombre.  El plan de ejecución del proyecto es un estudio de intervención dietética, controlado, randomizado, doble ciego y de tipo paralelo, con cuatro tipos distintos de bombones en pacientes afectos de hipercolesterolemia moderada. El período de estabilización será de 2 semanas y el período de intervención durará 4 semanas. La dieta de intervención será baja en grasa saturada e incluirá uno de los cuatro tipos de bombones. En concreto, los 4 tipos de bombones son: a) avellana; b) avellana y fitosteroles; c) avellana y componentes bioactivos ((fibra soluble e insoluble), ácido fólico, fitosteroles: denominado “portfolio”) y d) cacao que actuará como control. Cada pieza de bombón pesa unos 13 g y aporta unas 70 Kcal. Las dietas serán isocalóricas con la inclusión de las 6 piezas de bombones.  Si alguno de los bombones demuestra efectos beneficiosos sobre los factores de riesgo cardiovascular, este resultado permitirá que un bombón, clásico producto lúdico alimentario, apreciado por gran parte de la población, se pueda incluir en una dieta cardiosaluble. |
